# Anti-melanoma differentiation-associated gene 5 antibody associated rapidly progressive interstitial lung disease in a pediatric patient: a case report

**DOI:** 10.1186/s13256-025-05336-6

**Published:** 2025-06-13

**Authors:** Thitima Sirimontakan, Natalia Escobar, Fiona Kritzinger, Elizaveta Limenis, Greta Mastrangelo, Briseida Mema, Haifa Mtaweh

**Affiliations:** 1 Critical Care Medicine Department, The Hospital for Sick Children, Toronto, ON Canada; 2 The Interdepartmental Division of Critical Care Medicine, University of Toronto, Toronto, ON Canada; 3 Department of Pediatrics, University of Toronto, Toronto, ON Canada; 4 Division of Respiratory Medicine, The Hospital for Sick Children, Toronto, ON Canada; 5 Division of Rheumatology, The Hospital for Sick Children, Toronto, ON Canada

**Keywords:** Interstitial lung disease in children, Rapidly progressive interstitial lung disease, ILD, Anti-MDA5 antibody, Extracorporeal membrane oxygenation, ECMO, Case report, Juvenile dermatomyositis, JDM

## Abstract

**Background:**

Rapidly progressive interstitial lung disease presents as a severe complication of juvenile dermatomyositis, particularly when associated with anti-melanoma differentiation-associated gene 5. We report a pediatric case that underscores the necessity for clinicians to maintain a high index of suspicion for early identification and management.

**Case presentation:**

A previously healthy 7-year-old White girl presented with a 6-week history of generalized weakness, fever, joint pain, and abdominal pain. Initial examination revealed hypoxia, tachypnea, and hepatosplenomegaly. Laboratory tests were marked by thrombocytopenia, lymphopenia, elevated liver enzymes, high ferritin, high triglyceride, elevated muscle enzymes, and increased soluble IL-2 receptor, suggesting macrophage activation syndrome that was and managed with dexamethasone 5 mg/kg/m^2^ twice daily. There were no pathogenic skin features of juvenile dermatomyositis, except for nailfold capillary dropout. Initial cell counts revealed that her white blood cell count was 2.87 × 10^9^/L, hemoglobin was 105 g/L, platelet was 90 × 10^9^/L, and ferritin was 2000.6 μg/L and antinuclear and anti-Ro52 antibodies were positive. She was noted to have peripheral muscle weakness. Her clinical course was marked by progressive respiratory failure requiring mechanical ventilation with imaging revealing diffuse alveolar ground-glass opacities. The infectious work up was negative for bacterial, fungal, and viral ethologies including Epstein–Barr virus; hepatitis A virus, hepatitis B, hepatitis C, and hepatitis E viruses; parvovirus B19; cytomegalovirus; herpes simplex virus 1 and 2; and human herpesvirus 6. With the interstitial lung disease picture, pulse doses of intravenous methylprednisolone and intravenous immunoglobulin were initiated. She developed a significant air leak that was managed with bilateral chest tubes. Her significant hypoxemia required cannulation to veno-venous extracorporeal membrane oxygenation. The diagnosis of anti-melanoma differentiation-associated gene 5 antibody-associated juvenile dermatomyositis was confirmed by antibody testing. Additional immunomodulatory therapy was utilized during the treatment course with no noted improvement. She was not a candidate for lung transplantation, and in the face of additional organ dysfunction, life-sustaining therapies were withdrawn on day 32 of intensive care unit admission.

**Conclusions:**

This case demonstrates the diagnostic and therapeutic challenges in patients with rapidly progressive interstitial lung disease in the context of anti-melanoma differentiation-associated gene 5 associated juvenile dermatomyositis, who may not present with overt muscle and cutaneous features of juvenile dermatomyositis and whose lung disease can progress very rapidly. A high index of suspicion among clinicians is critical, and expedited diagnostic serology may assist with earlier diagnosis and initiation of therapy. Extracorporeal membrane oxygenation can be utilized as a bridge to diagnosis in the setting of severe refractory hypoxemic respiratory failure. However, despite aggressive treatment, the prognosis remains challenging.

## Background

Interstitial lung disease (ILD) in pediatric patients encompasses a broad spectrum of disorders characterized by inflammation and fibrosis of lung tissue. Factors that have been implicated in the development and progression of disease include genetic predisposition, environmental exposures, autoimmune disease, infections, and drug reactions [1, 2]. Children with ILD may present with cough, tachypnea, and unexplained hypoxia [3].

Rapidly progressive interstitial lung disease (RP-ILD) is a severe complication of juvenile dermatomyositis (JDM), particularly when associated with anti-melanoma differentiation-associated gene 5 (anti-MDA5) antibodies [2–5]. Physical examination findings may include tachypnea, bilateral crackles, and features of JDM, such as skin rash, ulcerative skin lesions, and fever, with or without muscle weakness [3, 4, 6]. Diagnostic tests such as pulmonary function testing; imaging including high-sensitivity computed tomography (CT) of the chest; and lung biopsy play a vital role in confirming the diagnosis [7]. Treatment of pediatric ILD involves anti-inflammatory and antifibrotics medications, along with supportive therapy such as oxygen and pulmonary rehabilitation to improve lung function. Disease prognosis depends on the underlying cause and the severity of lung damage, with complications such as pulmonary hypertension and respiratory failure potentially impacting long-term outcomes.

We present a distinctive case of anti-MDA5-antibody-associated RP-ILD in a child, highlighting the diagnostic challenges posed by the nonspecific nature of the presenting symptoms, and the therapeutic challenges related to disease severity and its rapid progression. This condition is rare, and there is a paucity of literature, largely comprised of case reports. Our case underscores the importance of maintaining a high index of suspicion to facilitate early identification and prompt management [3, 4, 6, 8–11].

## Case presentation

The legal guardians have provided informed consent for the preparation and publication of this case report.

A previously healthy, 7-year-old White girl presented with a 6-week history of vague, generalized weakness, fever, joint pain, and abdominal pain. There was no reported concern for familial diseases. She was admitted to the general pediatric ward with hypoxia, tachypnea, and hepatosplenomegaly. Initial laboratory tests revealed thrombocytopenia, lymphopenia, elevated liver enzymes, hyperferritinemia, hypertriglyceridemia, elevated soluble IL-2 receptors, and elevated muscle enzymes with a creatine kinase (CK) of 645 μ/L (Table 1). Infectious workup was not suggestive of an acute infection (Table 1). Macrophage activation syndrome (MAS) was suspected, and she was treated with dexamethasone 5 mg/kg/m^2^ twice daily. Due to increasing oxygen requirements, she was transferred to intensive care unit for noninvasive ventilation. Her clinical exam was marked by significant proximal muscle weakness and nailfold capillary dropout.

**Table 1:** First instance of tested laboratory values

Lab	Normal range	Results
Platelets	203–431 × 10^9^/L	90 × 10^9^/L
ALT	< 24 U/L	216 U/L
Ferritin	13.7–78.8 µg/L	2000.6 µg/L
Triglyceride	< 0.85 mmol/L	5.21 mmol/L
LDH	174–304 U/L	1119 U/L
CK	60–365 U/L	645 U/L
AST	< 42 U/L	982 U/L
ALT	≤ 24 U/L	216 U/L
Soluble IL-2 receptor	995–5253 pg/ml	9911 pg/ml
Anti-MDA-5 Ab	Negative	3 +
ANA		
Homogeneous		1:640
Speckled		1:640
Nuclear dot		1:320

Ab, antibodies; ALT, alanine transaminase; ANA, anitnuclear antibody; CK, creatine kinase; LDH, lactate dehydrogenase; Tx, treatment; VV-ECMO, veno-venous extracorporeal membrane oxygenation; ILD, interstitial lung disease; MAS, macrophage activation syndrome

**Fig. 1:**
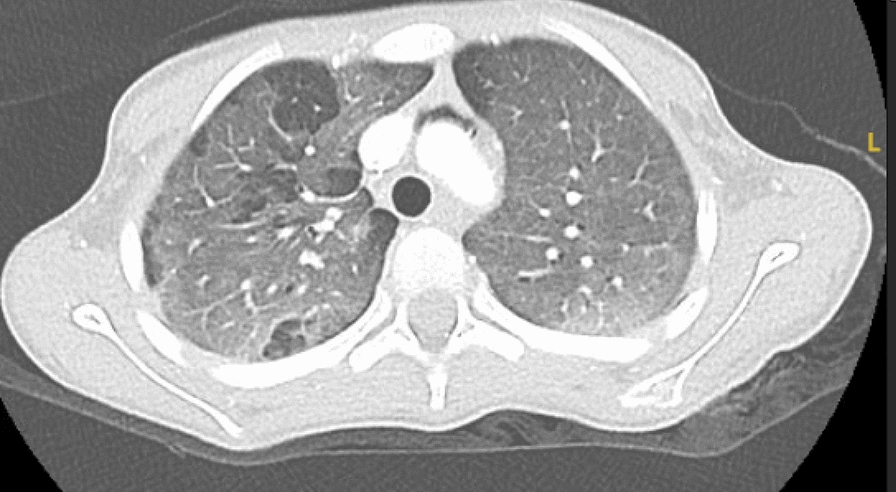
Diffuse alveolar ground-glass density in both upper lobes with sparing in upper lobes

**Fig. 2:**
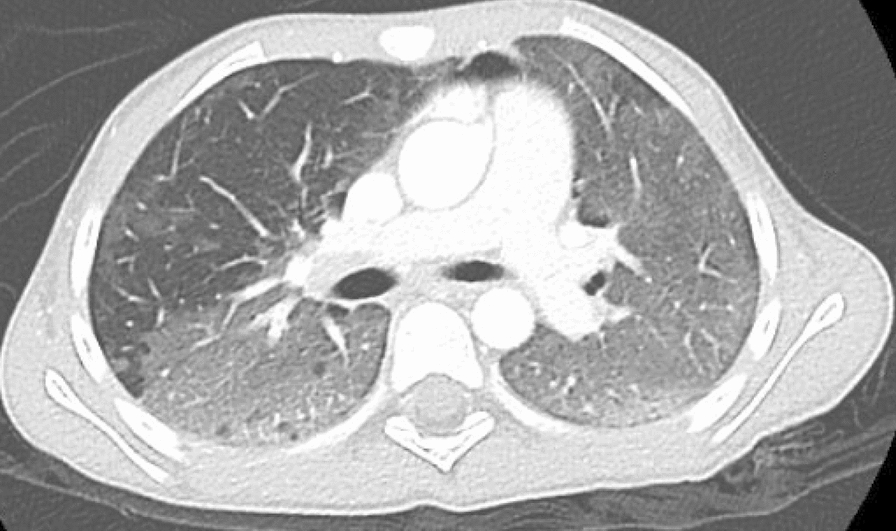
Diffuse ground-glass density with sparing in the right middle lobe

**Fig. 3:**
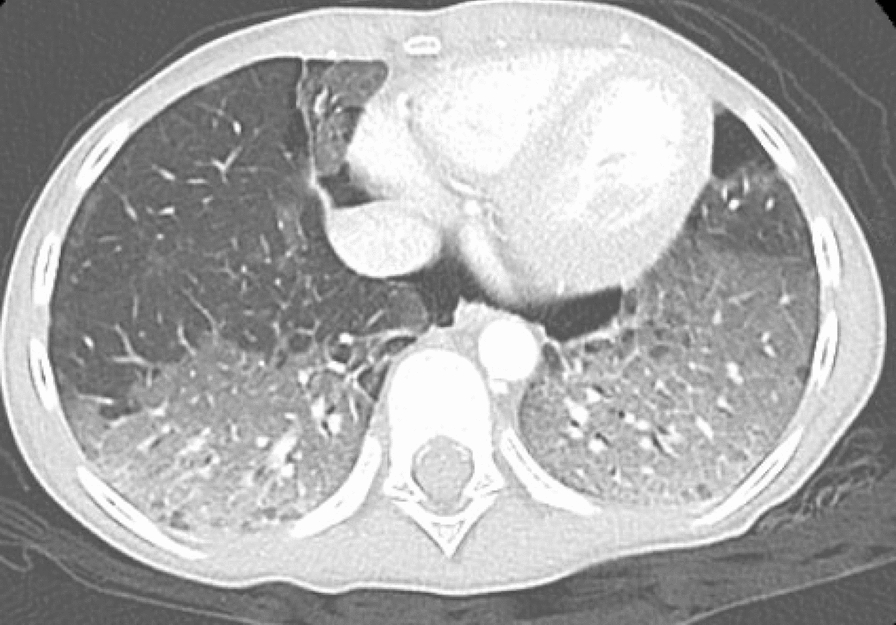
Bi-basal septal thickening and pneumomediastinum

Despite improvement in MAS markers and resolution of fever after initiation of corticosteroids, her respiratory state worsened, and she required intubation and mechanical ventilation. Her chest X-ray was suggestive of interstitial lung disease, and a chest CT confirmed diffuse alveolar ground-glass opacities (Figs. 1, 2), mild bi-basal septal thickening, and a moderate pneumomediastinum (Fig. 3). A bronchoscopy and bronchoalveolar lavage were performed and were negative for bacterial, viral, and fungal pathogens.

Due to the rapidly progressive respiratory failure and imaging features of interstitial lung disease, corticosteroids were escalated to pulse doses of intravenous methylprednisolone (30 mg/kg/day for 3 days), and intravenous immunoglobulin at a dose of 2 gm/kg was added. On day 2 after intubation, she developed a persistent air leak with significant hypoxemia despite drainage. She was cannulated to veno-venous (VV) extracorporeal membrane oxygenation (ECMO), and lung biopsy was undertaken, which showed diffuse alveolar damage in the organizing phase, patchy interstitial chronic inflammatory cell infiltrate, and viral inclusions suggestive of cytomegalovirus infection.

**Fig. 4:**
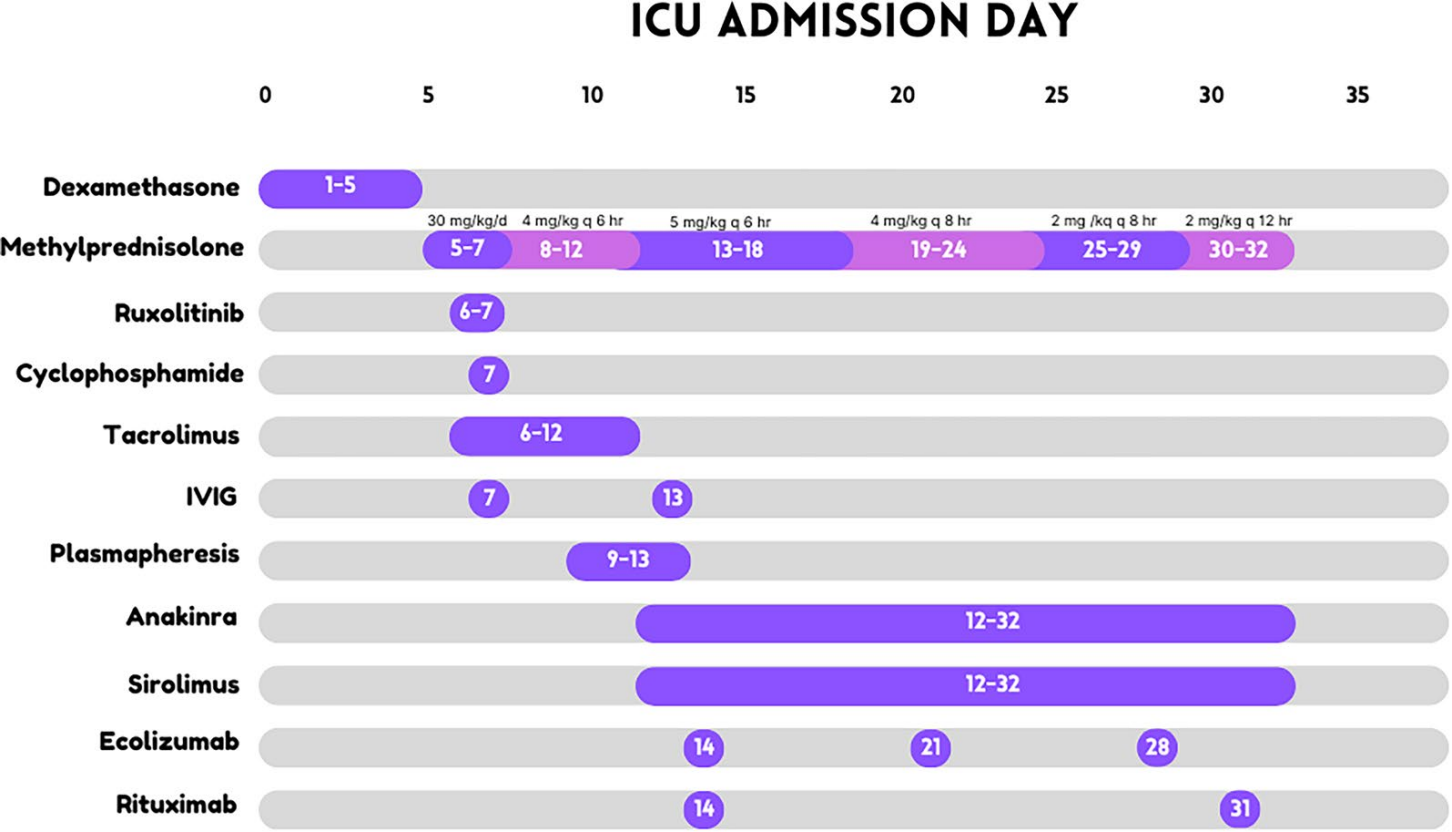
Timeline of administration of immunosuppressive agents

Shortly after cannulation to ECMO, anti-MDA5 antibody returned positive, confirming the diagnosis of anti-MDA5-antibody-associated juvenile dermatomyositis with RP-ILD phenotype. Despite the worsening respiratory state, CK continued to reduce, with levels reaching 55 µ/L by day 11. Additional immunomodulatory therapy was initiated at different time points including rituximab, tacrolimus, cyclophosphamide, plasma exchange, and anakinra (Fig. 4). Anakinra was used for the management of possible MAS/hyperinflammation when the ferritin rose from 800 to 7600 µ/L, with worsening thrombocytopenia, and hypofibrinogenemia while the patient was receiving high-dose corticosteroids. Eculizumab was later added due to elevated CH50 levels and complement staining on lung biopsy tissue.

**Fig. 5:**
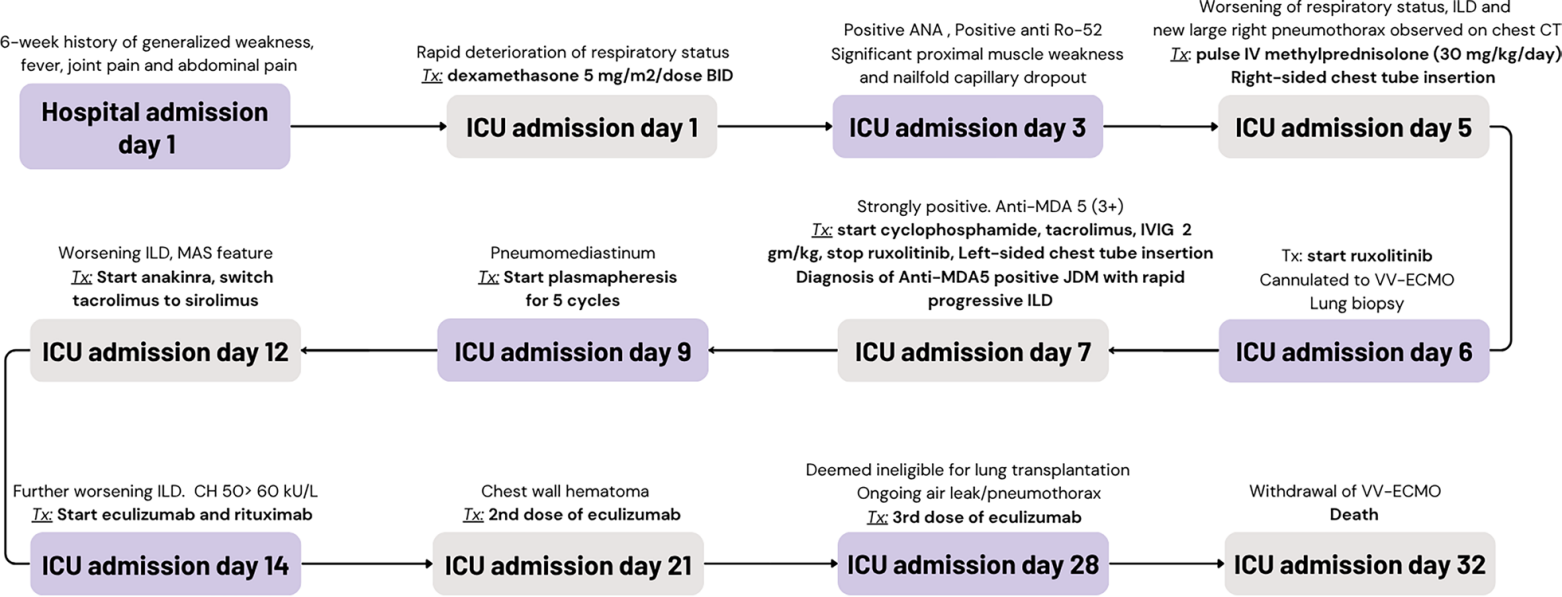
Treatment detail in chronological order

She remained on ECMO for 25 days. Her course was complicated by significant bleeding from the lung biopsy site and mucosal hemorrhage. With lack of improvement that would allow separation from ECMO, she was assessed for lung transplantation and was deemed unsuitable due to severe muscle wasting and deconditioning, which would preclude meaningful recovery. She developed additional organ dysfunction and was separated from life-sustaining therapy on hospital day 32. A timeline of her clinical course is presented in Fig. 5.

## Discussion

This case highlights the diagnostic challenges of interstitial lung disease associated with anti-MDA5-antibody-positive JDM, particularly in the absence of classical symptoms of JDM. It underscores the utility of early diagnostic serologic testing to enable prompt initiation of therapy, and illustrates the use of ECMO as a bridge to diagnosis in the setting of severe, refractory hypoxemic respiratory failure.

Anti-MDA5-associated JDM is a rare and severe form of the disease characterized by the presence of antibodies targeting MDA5, a protein involved in the immune response. The etiopathogenesis is thought to involve an unidentified viral trigger in genetically predisposed individuals, resulting in the development of an acquired type I interferonopathy [1]. Clinical features may include skin rash, ulcerative lesions, fever with or without muscle weakness, and frequent association with ILD, a condition marked by progressive lung fibrosis [1, 4, 12–15].

Studies have identified ethnic disparities in the severity of ILD in patients with anti-MDA5-antibody-positive dermatomyositis, with a lower rate of ILD reported among White individuals compared with East Asian populations. In North America and the UK, approximately 7% of JDM cases are anti-MDA5-positive, with 25% developing ILD and 5% experiencing a RP-ILD phenotype [14, 15]. In contrast, patient cohorts from Japan report a higher prevalence, with over one-third of JDM patients testing positive for anti-MDA5 antibodies, and more than half of those developing RP-ILD [2, 5]. RP-ILD is associated with a significantly worse prognosis, as seen in our patient [15, 16]. While MAS is rarely reported in anti-MDA5-associated JDM, it has been reported in adults with anti- MDA5-positive dermatomyositis [17].

Our case emphasizes the importance of recognizing this uncommon form of JDM and maintaining a high index of suspicion in patients exhibiting rapidly declining respiratory function, even in the absence of classic dermatomyositis features. In our patient, the only typical findings were nailfold capillary dropout and mild proximal muscle weakness. The diagnosis of JDM in our patient was supported by evidence from the chest CT supporting progressive interstitial lung disease with mediastinal emphysema, diffuse alveolar ground-glass opacities, and mild bibasilar septal thickening, which was then confirmed by the positive anti-MDA5 antibody test. The detection of myositis specific antibodies positive for MDA-5 was indicative of strong likelihood of JDM accompanied by rapidly progressive ILD.

Given the high mortality associated with anti-MDA5-positive JDM, a tailored therapeutic approach with planned and prompt intensification of therapy is necessary for those showing a poor response to initial therapy. This case underscores the need for early recognition and comprehensive evaluation of anti-MDA5-associated JDM, which can profoundly affect clinical outcomes. A proactive diagnostic and therapeutic strategy may reduce the need for invasive procedures, accelerate diagnosis, and improve care and quality of life. The incidence of anti-MDA5-associated RP-ILD in JDM is relatively rare, and standardized prevalence data are limited. Our literature review identified eight published pediatric cases of anti-MDA5-associated RP-ILD from seven articles, with one resulting in death [3, 4, 6, 8–11]. Anti-MDA5 antibodies have been strongly correlated with more severe ILD and increased morbidity and mortality.

In adult patients, current treatment strategies for anti-MDA5-associated ILD include using both glucocorticoids and a calcineurin inhibitor, or triple therapy with the additional of intravenous cyclophosphamide [18, 19]. A Japanese study reported six cases in which plasmapheresis was effective as an additional treatment in patients who did not respond to intensive immunosuppressive therapy [20]. Our patient received a combination of immunosuppressive medications including pulse dose intravenous methylprednisolone, rituximab (anti-CD 20 monoclonal antibody), cyclophosphamide, anakinra (interleukin-1 receptor antagonist), and plasma exchange. Additionally, due to persistent concerns over deteriorating lung function and complement staining on pathology from lung biopsy, the decision was made to administer both rituximab and eculizumab, human monoclonal antibodies that target complement C5. Our patient’s condition acutely deteriorated, and she was intubated, followed by cannulation onto VV-ECMO.

The utilization of ECMO for patients with RP-ILD has been well reported in adults but lacks sufficient description in pediatric cases. A case report from Japan described a case of anti-MDA5-antibody-positive JDM with RP-ILD in a patient who showed resistance to treatment with methylprednisolone, cyclosporin A, cyclophosphamide, immunoglobulin, and plasma exchange and was ultimately supported with VV-ECMO [11]. In another report, ECMO was used as a bridge to diagnosis in a child with interstitial lung disease who tested positive for the anti-MDA5 antibody and presented with clinically amyopathic dermatomyositis [21]. Despite receiving aggressive treatment, the prognosis remains guarded. The potential complication of these treatments can increase the risk of infection due to a diminished immune response. ECMO can be utilized as a bridge to diagnosis, treatment, or transplant if patient remains a candidate, or ultimately as a bridge to the decision for withdrawal of life-sustaining therapy.

Finally, the lack of targeted therapy for anti-MDA5-antibody-positive JDM with RP-ILD poses significant clinical difficulty. Current strategies for treatment are based on expert consensus. Further research is needed to identify effective therapies and improve outcomes in this high-risk population.

## Conclusion

Anti-MDA5-positive JDM with associated ILD presents a unique challenge to clinicians particularly in the absence of classical features of JDM. A high index of suspicion is critical in patients displaying rapid decline in lung function, and early serologic testing can assist in expediting diagnosis and treatment. Further research is needed to determine the most effective treatment for this subset of JDM patients, due to the poor prognosis associated with the disease.

## Data Availability

None available.
